# TAL1 overexpression in induced pluripotent stem cells promotes the formation of hematopoietic cell-forming complexes but inhibits enucleation *in vitro*

**DOI:** 10.3389/fcell.2025.1474631

**Published:** 2025-04-24

**Authors:** Arim Shin, Sung Ah Park, Ji Yeon Kim, Eun Jung Baek

**Affiliations:** ^1^ Department of Translational Medicine, Graduate School of Biomedical Science and Engineering, Hanyang University, Seoul, Republic of Korea; ^2^ Department of Laboratory Medicine, College of Medicine, Hanyang University, Seoul, Republic of Korea; ^3^ ArtBlood Inc., Seoul, Republic of Korea

**Keywords:** induced pluripotent stem cells, *In vitro* erythropoiesis, hematopoietic cell-forming complex, enucleation, TAL1

## Abstract

The *in vitro* generation of human red blood cells (RBCs) from stem cells, such as induced pluripotent stem cells (iPSCs), holds promise for transfusable RBCs but faces challenges, including RBC maturation, enucleation, and large-scale production. In this study, we evaluated the effect of conditional TAL1 overexpression on *in vitro* RBC production via hematopoietic cell-forming complex (HCFC) formation from iPSCs because *TAL1* is a key regulatory transcription factor essential for erythropoiesis. *TAL1* overexpression in iPSCs, either before or after hematopoietic induction, significantly enhanced HCFC formation and hematopoietic differentiation, as evidenced by increased hematopoiesis-related gene expression, a higher yield of glycophorin A (GPA)+/CD71+ cells, and elevated gamma hemoglobin levels. These findings highlight the potential of TAL1 as a powerful regulator of erythropoiesis *in vitro* and offer a promising strategy for improving RBC production from stem cells. However, the reduced enucleation efficiency observed after TAL1 overexpression indicates a key challenge that must be addressed to optimize the generation of fully functional, transfusable RBCs. Further research is required to balance the benefits of enhanced differentiation with the need for efficient enucleation, which is critical for the production of mature, viable RBCs.

## 1 Introduction

Red blood cell (RBC) transfusion is critical to clinical care. *In vitro* RBC production has the potential to address supply shortages and provide contamination-free cells, thus becoming a standard procedure in the future. Initially, CD34^+^ cells from cord blood (CB), peripheral blood (PB), and bone marrow were used to generate RBCs *ex vivo* (Lee et al., 2018). However, the limited expansion capacity of CD34^+^ cells and the difficulty of obtaining early-stage CD34^+^ cells have led to the exploration of induced pluripotent stem cells (iPSCs) as alternatives. Consequently, various approaches have been developed to differentiate iPSCs into RBCs (Bernecker et al., 2019; Kurita et al., 2013; Uchida et al., 2017; Haro-Mora et al., 2020; Kobari et al., 2012; Olivier et al., 2016; Sivalingam et al., 2018; Demirci et al., 2020; Sivalingam et al., 2021; Ying Wang et al., 2016). However, this process mimics primitive erythropoiesis, and the enucleation rate of iPSC-derived RBCs remains below 30%. Various active and alternative strategies have been explored to overcome this challenge, produce definitive RBCs, and improve erythroid differentiation and enucleation efficiency. These include incorporating additional feeder cells during differentiation to enhance enucleation (Sivalingam et al., 2021).

In a recent study, Dorn et al. Bernecker et al. (2019) successfully generated RBCs from iPSCs expressing fetal hemoglobin and exhibiting a high enucleation rate. This was achieved through an embryoid body (EB)-mediated differentiation process mimicking definitive erythropoiesis. A key feature of this process is the formation of a hematopoietic cell-forming complex (HCFC), which includes naturally occurring stromal cells that emerge without artificial feeder layers. As our tests revealed, a major limitation of this approach is that only 19% of colonies differentiated from EBs during hematopoietic induction ultimately form HCFC (see Figure 3C). The overexpression (OE) of additional transcription factors involved in hematopoietic progression may offer a promising strategy to address this limitation and enhance HCFC formation.

Several transcription factors have been identified as critical regulators of hematopoietic progression. Studies have shown that introducing a combination of *GATA2*, *TAL1*, *LMO2*, and *ETV2* into iPSCs enhances their hematopoietic differentiation potential (Elcheva et al., 2014). Similarly, the addition of *Erg*, *Fli1*, *Tal1*, *Lyl1*, *Lmo2*, *Runx1*, *Cbfb*, and *Gata2* promotes the generation of hematopoietic progenitor cells in mouse embryonic stem cells (ESCs) (Bergiers et al., 2018).

Among these transcription factors, *TAL1* is a master regulatory factor crucial for establishing both primitive and definitive hematopoiesis. Evidence from *Tal1* knockout murine models has revealed a failure in both primitive and definitive erythropoiesis during embryonic development (Robb et al., 1996; Robb et al., 1995). Additionally, the conditional deletion of *Tal1* resulted in impaired maturation of erythroid cells, although differentiation into hematopoietic cells is not affected (Schlaeger et al., 2005). Conversely, the OE of *Tal1* in zebrafish embryos increases blood and endothelial tissue formation (Dooley et al., 2005). Furthermore, forced expression of TAL1 in iPSCs significantly enhanced the efficiency of hematopoietic cell induction (Kurita et al., 2013).

Despite advances in RBC production using iPSCs, the efficiency of HCFC formation and enucleation remains suboptimal, hindering clinical applications. This study seeks to address these gaps by exploring the role of TAL1 in improving hematopoietic progression and HCFC formation.

## 2 Materials and methods

### 2.1 CB-mononuclear cell (CB-MNC) isolation

Umbilical cord blood was donated after obtaining written informed consent from healthy mothers who agreed to donate. This study was approved by the Institutional Review Board of Hanyang University (IRB No. HYI-2019-07-006). CB-MNCs were cultured after isolation by centrifugation using Ficoll–Paque (Cytiva, Marlborough, MA, United States).

### 2.2 Generation of CB-MNC-derived iPSCs

CB-MNCs were cultured in Iscove’s modified Dulbecco’s medium (IMDM; Gibco, Waltham, MA, United States) supplemented with 200 μg/mL holo-transferrin (Sigma-Aldrich, St. Louis, MO, United States), 10 μg/mL insulin (Sigma-Aldrich), 3 IU/mL heparin (Sigma-Aldrich), and 6 IU/mL erythropoietin (EPO; Calbiochem, San Diego, CA, United States), 100 ng/mL stem cell factor (SCF; R&D Systems, Minneapolis, MN, United States), and 10 ng/mL interleukin-3 (IL-3; R&D Systems) in the presence of 5% fetal bovine serum (Hyclon, Logan, UT, United States) and 1% penicillin–streptomycin (Gibco). CB-MNCs differentiated into erythroblasts 6–7 days prior to iPSC reprogramming (Supplementary Figure S1). Subsequently, erythroblasts were electroporated for transfection with the Neon Transfection System (1,650 V, 10 ms, three pulses) using the Epi5^TM^ Episomal iPSCs Reprogramming vector (Invitrogen, Carlsbad, CA, United States). Transfected erythroblasts were seeded in six-well tissue culture plates (Nunc, Waltham, MA, United States) coated with hESC-qualified Matrigel (Corning, NY, United States). When iPSC-like colonies were observed while culturing in ReproTeSR^TM^ Medium (StemCell Technologies, Vancouver, Canada), only reprogrammed colonies were selected through TRA 1-60 live cell staining (Invitrogen).

### 2.3 iPSC culture

Reprogrammed colonies were maintained on plates coated with Matrigel. iPSCs were cultured in mTeSR^TM^ 1 medium (StemCell Technologies) in a 37°C, 5% CO_2_ incubator until the cells reached 70% confluency. The iPSCs were passaged every 5–7 days using ReLeSR^TM^ (StemCell Technologies), and the culture medium was replaced every 2 days.

### 2.4 Transduction of *TAL1* into iPSCs

The lentiviral vector FUW-tetO-MCS (Addgene, Watertown, MA, United States) was cloned into the synthesized *TAL1* gene using the EcoR1 restriction site. *GFP* was synthesized and inserted into the vector using the BamH1 restriction site to produce FUW-tetO-TAL1-GFP. *TAL1* and *GFP* were linked via the T2A peptide. Lentiviruses were generated from 293FT cells. On the day of passage, iPSCs were transduced with the lentiviruses at a multiplicity of infection (MOI) of 10 in mTeSR^TM^ 1 medium supplemented with 2 μg/mL polybrene (Sigma). After 24 h, the culture medium was replaced with a fresh mTeSR^TM^ 1 medium containing 1 μg/mL doxycycline (Takara, Shiga, Japan). For the transduced iPSCs, only colonies expressing GFP were manually selected (Zhu et al., 2023).

### 2.5 HCFC induction

The HCFC was induced using a modified version of a previously described method (Bernecker et al., 2019). iPSCs were detached into large clumps using ReLeSR. EBs were formed using the AggreWell^TM^ EB formation medium (StemCell Technologies). To promote HCFC formation, EBs were cultured in STEMdiff^TM^ APEL^TM^ 2 medium (STEMCELL Technologies) supplemented with 3 IU/mL EPO, 100 ng/mL SCF, 5 ng/mL IL-3, and 1 μg/mL doxycycline, along with 5% protein-free hybridoma medium II (Gibco) and 1% penicillin–streptomycin, in 12-well tissue culture plates (Nunc). The culture medium was replaced weekly, and non-adherent EBs were removed 2–3 weeks after induction.

### 2.6 Erythroid differentiation

**FIGURE 1:**
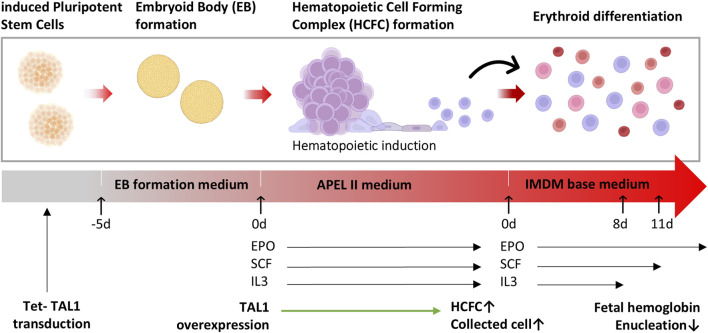
Differentiation of hematopoietic cell-forming complexes (HCFC) through embryoid bodies (EBs). A schematic representation of the differentiation process of induced pluripotent stem cells (iPSCs) to erythrocytes through the formation of EBs and HCFCs. iPSCs were dissociated and cultured in ultra-low binding plates to form EBs. After 5 days, the EBs were transferred into hematopoietic induction media. Upon the formation of HCFCs, the dissociated cells were cultured in differentiation media and subsequently harvested as erythrocytes.

HCFC cells were cultured in the medium supplemented with 10% human serum (Sigma-Aldrich), 330 μg/mL holo-transferrin, 10 μg/mL insulin, and 1% penicillin–streptomycin in IMDM, and the detached cells in the culture medium were collected every week and differentiated through three stages of erythroid differentiation up to day 18 (Figure 1). On days 0–8 of the differentiation culture, 3 IU/mL EPO, 100 ng/mL SCF, and 5 ng/mL IL-3 were added. On days 8–11 of the differentiation culture, 3 IU/mL of EPO and 100 ng/mL of SCF were added. From days 11 to 18, only 3 IU/mL of EPO was supplemented.

### 2.7 Statistical analysis

All data from three or more independent experiments are presented as mean ± standard error. Statistical analyses were performed using R Programming 4.1.0 and GraphPad Prism 9.4.1, employing the non-parametric Student’s t-test and Wilcoxon signed-rank test. Statistical significance was defined as a p-value of 0.05 or less.

## 3 Results

### 3.1 HCFC is a precursor population before the endothelial-to-hematopoietic transition

Although iPSCs can be generated from various cell types, CB was chosen for this study because of its accessibility and higher proportion of CD34^+^ cells than PB. In addition, iPSCs derived from hematopoietic cells exhibit a greater propensity to differentiate into hematopoietic lineages (Dorn et al., 2015). Thus, to evaluate the *in vitro* erythroid differentiation potential of iPSCs, two iPSC lines were established using Yamanaka factors from CB-MNCs (Supplementary Figure S1). Notably, one line was derived from a male donor and the other from a female donor (Supplementary Figure S2), enabling a reduction in differentiation discrepancies between iPSC clones and sexes during the RBC differentiation process.

iPSCs differentiate into the erythroid lineage through a three-stage process: EB formation, hematopoietic induction, and erythroid differentiation, as described by Bernecker et al. (2019) (Figure 1).

**FIGURE 2:**
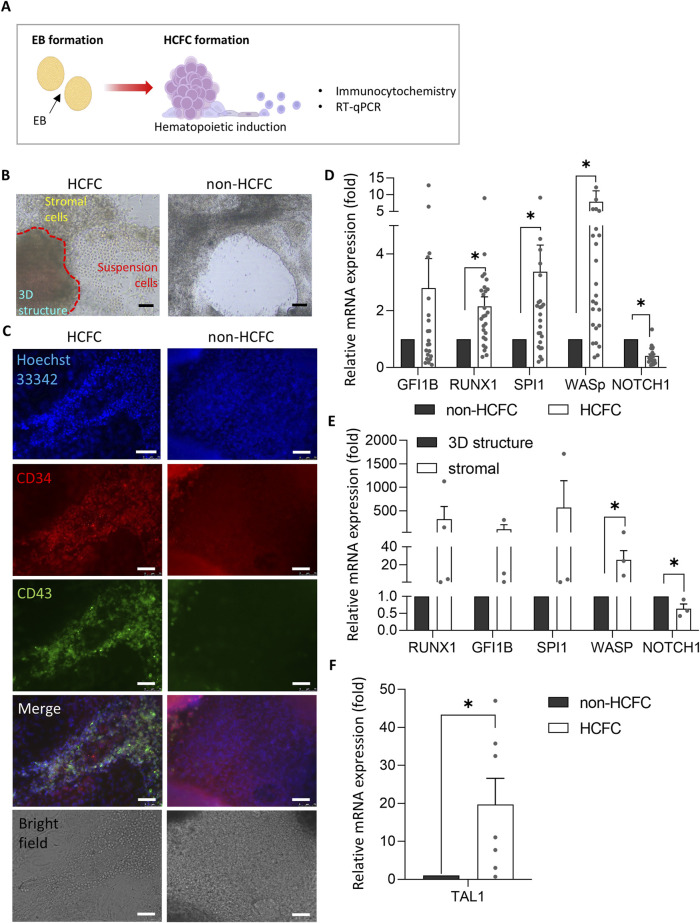
Characterization of HCFC: fluorescence and gene expression analysis. **(A)** Schematic representation of EB and HCFC formation during hematopoietic induction. **(B)** Inverted microscope images of HCFC and non-HCFC colonies show HCFC colonies releasing cells into the supernatant. Scale bar = 75 μm. **(C)** Immunofluorescence imaging of HCFC-released cells and non-HCFC colonies using a confocal microscope (n = 2). Hoechst 33342 (blue, nuclei), CD34 (red, hematopoietic stem cells), and CD43 (green, hematopoietic cells). Colonies shown were cultured in separate wells in a single experiment. Scale bar = 75 μm. **(D)** Gene expression analysis of hematopoiesis-related (*GFI1B*, *RUNX1*, *SPI1*, and *WASp*) and endothelial cell-associated (*NOTCH1*) genes in HCFC (n = 14) and non-HCFC (n = 6) using RT-qPCR. Data are presented as relative quantification (2^−ΔΔCT^). Statistical significance: *p < 0.05. **(E)** Comparison of hematopoiesis-related (*GFI1B*, *RUNX1*, *SPI1*, and *WASp*) and endothelial cell-associated (*NOTCH1*) gene expression between stromal cells and 3D structures within HCFCs (n = 4) by RT-qPCR. Each colony was considered an individual sample. *p < 0.05. **(F)** *TAL1* expression analysis in HCFC (n = 7) and non-HCFC (n = 3) by RT-qPCR. *p < 0.05. Statistical analyses were performed using Student’s t-test (for E and F) and Wilcoxon’s signed-rank test (for D). Data are presented as mean ± SEM. HCFC, hematopoietic cell-forming complex.

**FIGURE 3:**
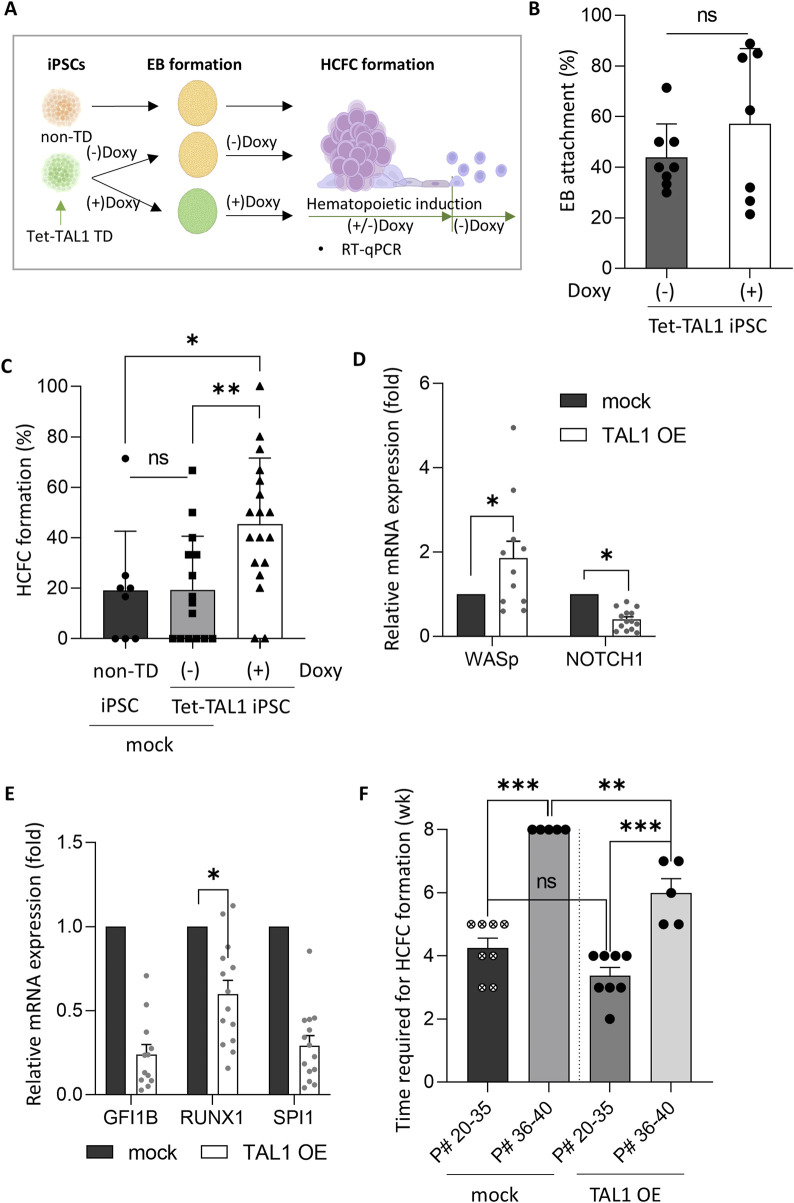
TAL1 OE enhances hematopoietic induction. **(A)** Schematic representation of the timing of TAL1 OE during hematopoietic induction, achieved by adding doxycycline from the iPSC stage to HCFC formation. **(B)** Assessment of the effect of TAL1 OE timing on the EB adhesion rate in TAL1-OE iPSC colonies. TAL1 OE was initiated either at the iPSC stage or immediately after EB attachment (*Doxy(+)*, n = 3; *Doxy(−)*, n = 5). **(C)** Impact of TAL1 OE on HCFC formation. HCFCs were visualized using an inverted microscope, and the HCFC formation rate was calculated as the ratio of HCFCs to total colonies. *p < 0.05, **p < 0.01 (*non-TD*, n = 8; *Doxy(−)*, n = 15; *Doxy(+)*, n = 18). **(D, E)** Effect of TAL1 OE on the expression of hematopoiesis- and endothelial differentiation-related genes. mRNA expression levels were measured by RT-qPCR and presented as relative quantification (2^−ΔΔCT^). *p < 0.05 (mock, n = 4; TAL1 OE, n = 4). **(F)** Time required for HCFC formation based on the iPSC passage number. **p < 0.01; ***p < 0.001. Statistical analyses were performed using Student’s t-test (for C) and Wilcoxon’s signed-rank test (for D, F). Data are presented as mean ± SEM.

During the hematopoietic induction stage, HCFCs were characterized using immunocytochemistry and RT-qPCR (Figure 2A). After approximately 3–4 weeks, the cells within the EBs undergo hematopoietic induction, producing HCFC, which subsequently releases cells into the supernatant (Figure 2B). During hematopoietic induction, only 19% of the colonies from the embryoid bodies differentiate into HCFCs, resulting in a low yield of released cells in the culture medium (see Figure 3C).

Immunofluorescence staining revealed distinct expression patterns in HCFCs and non-HCFCs (Figure 2C). HCFCs exhibited the co-localization of CD34 and CD43, whereas non-HCFCs displayed low CD43 expression, even though CD34 was expressed. This suggests that in non-HCFCs, differentiation progressed only to the hemogenic endothelium stage and did not advance to HCFC formation. HCFCs showed robust expression of hematopoietic markers, particularly *WASp*, whereas non-HCFCs showed elevated *NOTCH1* expression (Figure 2D).

Next, we investigated which part of the HCFC differentiated into suspension cells. HCFCs were divided into two distinct populations: stromal cells, which adhered to the bottom of the culture plate, and 3D structures, characterized by cells that formed thick or spherical shapes, expanding into three-dimensional formations (Figure 2B). We hypothesized that the transition from 3D structures to stromal cells involves molecular and cellular changes, specifically an endothelial-to-hematopoietic transition (EHT), similar to the aorta–gonad–mesonephros (AGM) region (Zeng et al., 2019). During EHT, endothelial gene expression decreases, whereas hematopoietic gene expression gradually increases (Guibentif et al., 2017).

Our analysis revealed that stromal cells exhibited higher expression levels of hematopoiesis-related genes than 3D structures (Figure 2E). These findings indicate that HCFCs are in a transitional phase before undergoing EHT and suggest that EHT primarily occurs in stromal cells rather than in 3D structures. The elevated expression of endothelial cell-associated *NOTCH1*, especially in 3D structures, further supported this hypothesis.

### 3.2 *TAL1* OE enhances HCFC formation

As the HCFC formation rates in our iPSC lines were low (approximately 19%, as mentioned above), additional measures to enhance HCFC formation rates are recommended. Such improvements are essential if the HCFC-mediated hematopoietic progression of iPSCs can be effectively utilized for practical *in vitro* RBC production. We hypothesized that the OE of *TAL1*, among the various key regulators of hematopoietic progression, would enhance HCFC-mediated hematopoietic progression of iPSCs, as mentioned in the Introduction. Interestingly, *TAL1* expression in HCFCs was measured to be 20 times higher than in non-HCFCs at week 7 of hematopoietic induction (Figure 2F). Based on this finding, to improve HCFC formation and RBC differentiation, we tested *TAL1* OE using a Tet-inducible system to selectively express TAL1 at specific stages (Figure 3A). Lentiviruses containing the Tet-inducible FUW-tetO-TAL1-GFP construct were transduced into iPSCs, and *TAL1*-transduced iPSCs (Tet-TAL1 iPSCs) were isolated by manually selecting GFP-positive iPSC colonies (Supplementary Figure S3A). Tet-TAL1 iPSCs exhibited an 8-fold increase in *TAL1* transcript levels following doxycycline treatment (Supplementary Figure S3B).

We used a Tet-inducible expression system (Tet-On) in this study. Although this system was previously optimized, we were concerned about potential leakiness in the Tet-On system. A comparison between non-transduced iPSCs and non-doxycycline-treated tet-TAL1 iPSCs as a control group revealed no significant difference in the HCFC formation rates (Figures 3A, C; Supplementary Figure S5).

To determine whether the timing of *TAL1* OE affects EB formation efficiency, we overexpressed TAL1 either in iPSCs or immediately after EB attachment. No differences were observed in the timing of OE. Notably, in the *TAL1*-OE group, the HCFC formation rate significantly increased from 19% to 45%, although no difference was observed in the EB attachment rate, regardless of *TAL1* OE (Figures 3B, C). Gene expression analysis revealed elevated levels of hematopoiesis-related genes in the *TAL1*-OE group, with a significant upregulation of *WASp* and downregulation of endothelial cell-associated *NOTCH1* compared to the mock group (Figure 3D). Additionally, the expression of other hematopoiesis-related genes—GFI1B, SPI1, and RUNX1—showed stage-dependent variation, which may account for observed fluctuations in gene expression (Guibentif et al., 2017) (Figure 3E).

Notably, HCFC formation was delayed and occurred at a reduced rate as the passage number of iPSCs increased (Figure 3F). Specifically, when the passage number was below 35, HCFCs formed within 4 weeks. However, as the passage number approached 40, the HCFCs did not form. To investigate whether TAL1 OE in iPSCs could accelerate HCFC formation, we compared TAL1 OE and mock groups. No significant differences were observed in the time required for HCFC formation when the passage number was below 35. However, HCFCs appeared within 6 weeks in TAL1 OE iPSCs with passage numbers exceeding 40 (Figure 3F). These data suggest that *TAL1* OE is a prerequisite for efficient HCFC formation during hematopoietic cell induction, although we did not measure TAL1 expression in aged iPSCs.

### 3.3 *TAL1* OE accelerated the induction of erythroid lineage differentiation

**FIGURE 4:**
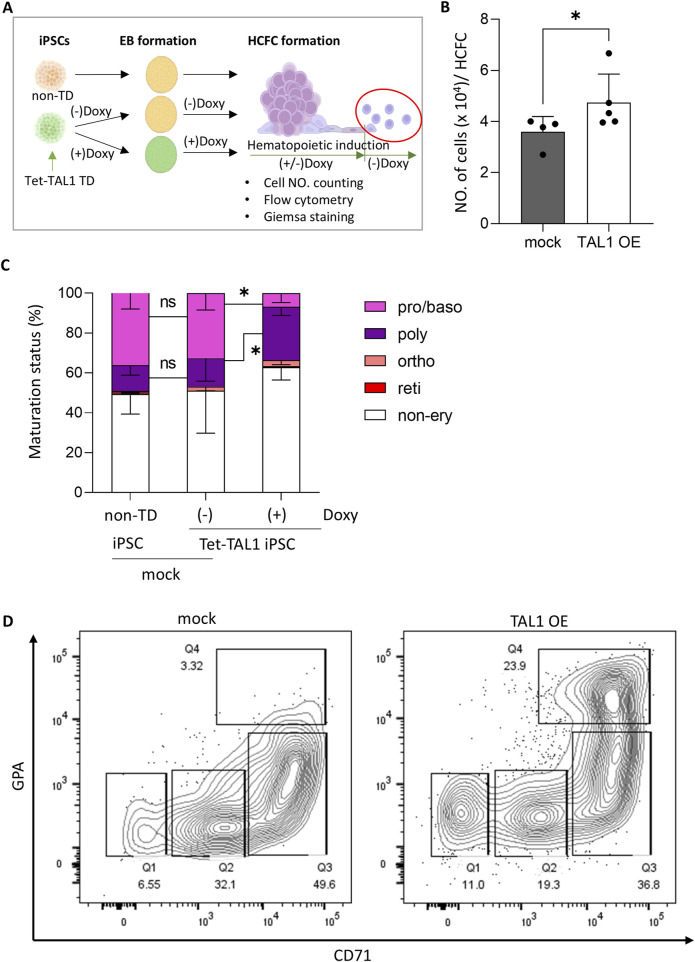
TAL1 OE accelerates early stages of erythroid differentiation after hematopoietic induction. **(A)** Schematic illustrating the experimental design. TAL1 OE was terminated midway through hematopoietic induction by removing doxycycline, and the released cells were further differentiated into the erythroid lineage. **(B)** Effect of TAL1 OE on the maximum number of cells harvested weekly from a single HCFC. Cell counts were performed using a hemocytometer. *p < 0.05 (mock, n = 4; TAL1 OE, n = 5). **(C)** Proportions of cells at each erythroid differentiation stage among those released from HCFCs at week 5, when the highest numbers of cells were released. Morphological evaluation was performed on 100–300 cells per condition using Wright–Giemsa staining. *p < 0.05 (*non-TD*, n = 6; *Doxy(−)*, n = 3; *Doxy(+)*, n = 6). **(D)** Flow cytometry analysis of cells released from HCFCs at 5 weeks during hematopoietic induction, stained for GPA and CD71. Quadrants: Q1 (CD71^−^/GPA^−^), Q2 (CD71^dim^/GPA^−^), Q3 (CD71^+^/GPA^dim^), Q4 (CD71^+^/GPA^+^). Statistical significances were analyzed using Wilcoxon’s signed-rank test. Data are shown as mean ± SEM. HCFC, hematopoietic cell-forming complex.

Next, we assessed the erythroid lineage differentiation potential of *TAL1*-overexpressing HCFCs by characterizing erythroid lineage cell types (Figure 4A). When we counted the number of cells per HCFC in the conditional *TAL1* overexpressing Tet-TAL1 iPSCs, where doxycycline was added from iPSCs to hematopoietic induction, the maximum cell yield per week from a single HCFC was significantly higher in the TAL1-OE group (4.7 × 10^4^ ± 0.6 × 10^4^) than in the mock group (3.6 × 10^4^ ± 1.1 × 10^4^) (Figure 4B).

To observe the impact of early *TAL1* OE during hematopoietic induction, we examined the cell types released from HCFC derived from non-transduced iPSCs and Tet-TAL1 iPSCs cultured in the presence or absence of doxycycline. In all groups, the number of cells collected from the supernatant was initially low during the early weeks, peaked at weeks 5–6, and then declined. Therefore, we collected the released cells at week 5 of hematopoietic induction and characterized the cell types using Wright–Giemsa staining (Supplementary Figure S4). In groups of non-transduced iPSCs and Tet-TAL1 iPSCs cultured in the absence of doxycycline, the ratio of erythroid lineage cells to non-erythroid cells was approximately 1:1, whereas, in the TAL1-OE group, the proportion of erythroid lineage cells decreased to approximately 40% (Figure 4C).

As terminal erythroid lineage differentiation leads to enucleated RBCs via sequential morphological and functional transitions of proerythroblasts and basophilic, polychromatophilic, and orthochromatic erythroblasts, which are characterized by increased hemoglobin accumulation and subsequent chromatin condensation, we could distinguish cells at specific stages of differentiation using Wright–Giemsa staining. Most erythroid lineage cells were in the pro/basophilic or polychromatophilic stages in all the groups. However, while the pro/basophilic erythroblast population comprised approximately 70% of the cells in the mock group, a higher proportion of polychromatophilic erythroblasts, comprising approximately 80% of the cell population, appeared in the TAL1-OE group, along with a greater presence of a more differentiated orthochromic population (Figure 4C).

As shown by the flow cytometry results, the TAL1-OE group exhibited a glycophorin A (GPA)+/CD71+ population of up to 24%, compared to only 3.5% in the mock group (Figure 4D). These findings suggest that early *TAL1* OE facilitates erythroid differentiation into more advanced erythroblasts, although other hematopoietic differentiations may also occur more efficiently.

### 3.4 *TAL1* OE inhibits erythroblast enucleation

**FIGURE 5:**
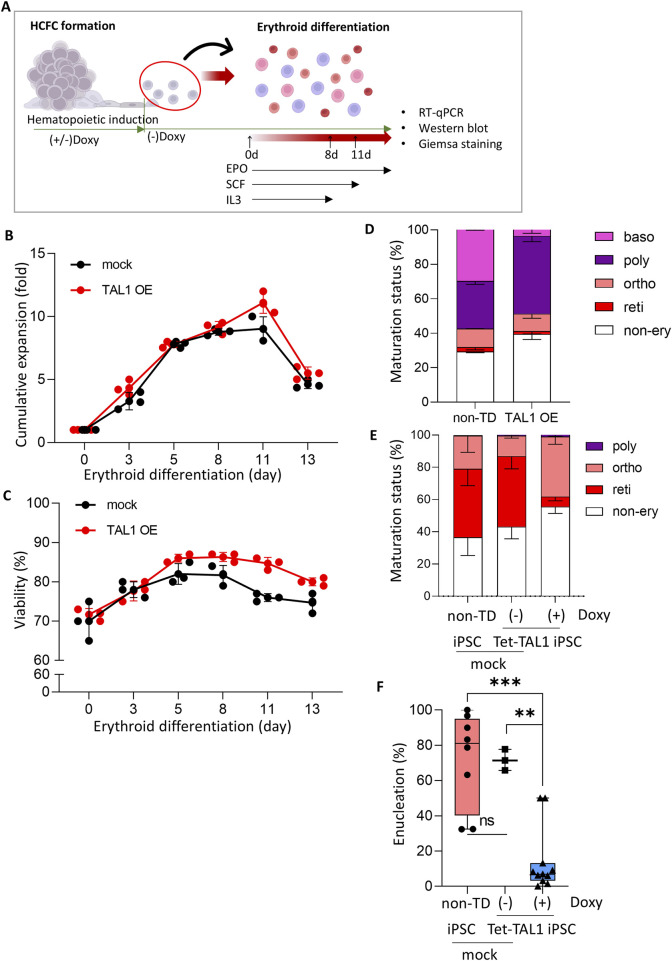
Effects of TAL1 OE during hematopoietic induction on terminal erythroid differentiation. **(A)** Schematic illustrating the experimental design. Cells released at weeks 3–7 from the hematopoietic induction were used for terminal erythroid differentiation. **(B, C)** Cumulative fold expansion **(B)** and cell viability **(C)** of cells harvested from HCFC during erythroid differentiation (mock, n = 3; TAL1 OE, n = 3). **(D, E)** RBC maturation status of cells at day 8 **(D)** and day 13 **(E)** of terminal differentiation. **(F)** Enucleation rate in cells at day 13 of terminal differentiation. A total of 50–300 cells per condition were counted. Plots show the median with min/max values. ***p < 0.001.

Lastly, we accessed the effect of *TAL1* OE on terminal erythroid differentiation by cultivating cells released from HCFC during the three stages of erythroid differentiation. Over a 2-week period, we supplemented the cultures with stage-specific cytokines and analyzed the molecular and cellular characteristics of the cells (Figure 5A).

Notably, we removed doxycycline midway through the hematopoietic induction process to minimize the effects of TAL1 OE during erythroid differentiation. TAL1 plays a critical role in the EHT and the activation of the hematopoietic transcriptional program (Vagapova et al., 2018). However, TAL1 expression levels vary during erythroid differentiation, and constitutive TAL1 expression in HSCs causes a differentiation imbalance, favoring myeloid over lymphoid cell production *in vivo* during xenotransplantation (Reynaud et al., 2005). Furthermore, uncontrolled TAL1 expression is associated with T-cell acute lymphoblastic leukemia in humans (Ferrando et al., 2002).

Based on these findings, we decided not to overexpress TAL1 throughout erythroid differentiation, as sustained high TAL1 expression may not be beneficial. Therefore, doxycycline, which was initially added to induce TAL1 expression in TAL1-OE iPSCs, was removed after the emergence of HCFCs. Following doxycycline withdrawal, most TAL1-OE cells exhibited reduced TAL1 expression and no longer displayed GFP fluorescence (Supplementary Figure S5).

Under these conditions, the proliferation rate and viability of the cells undergoing erythroid differentiation were unaffected by TAL1 OE (Figures 5B, C). This indicated that prior TAL1 OE did not influence the ongoing differentiation of erythroid cells.

We measured the maturation status of cells on days 8 (i.e., after the first phase of the differentiation process) and 13 (i.e., after completion of the terminal differentiation process) of erythroid differentiation by Wright–Giemsa staining (Supplementary Figure S6). The proportions of erythroid cell types on day 8 did not differ from those of cells released from the HCFC in both the mock and Doxy (+) Tet-TAL1 iPSCs groups (Figure 5D; compare also with Figure 4C). Importantly, compared to non-transduced iPSCs and Tet-TAL1 iPSCs cultured without doxycycline, Doxy (+) Tet-TAL1 iPSCs at day 13 exhibited significant inhibition of terminal erythroid differentiation, showing the highest proportion of cells at the orthochromatic erythroblast stage and an increased proportion of non-erythroid lineage cells (Figure 5E). In both non-transduced iPSC and doxycycline-free Tet-TAL1 iPSC, the ratio of erythroid lineage cells to non-erythroid cells was approximately 3:2. However, in the doxycycline-treated Tet-TAL1 iPSCs, the proportion of erythroid lineage cells decreased by approximately 45%. Furthermore, according to Wright–Giemsa staining, in doxycycline-free Tet-TAL1 iPSCs, approximately 40% of the cells were at the orthochromatic stage (Figure 5E), but the enucleation rate was low at only 15% (Figure 5F). In contrast, cells in both mock groups exhibited a much higher enucleation rate of approximately 60%–70%, and most cells were arrested at the orthochromatic stage (Figures 5E, F). These data suggest that TAL1 OE during hematopoietic induction impairs terminal erythroid differentiation, significantly reducing enucleation efficiency.

**FIGURE 6:**
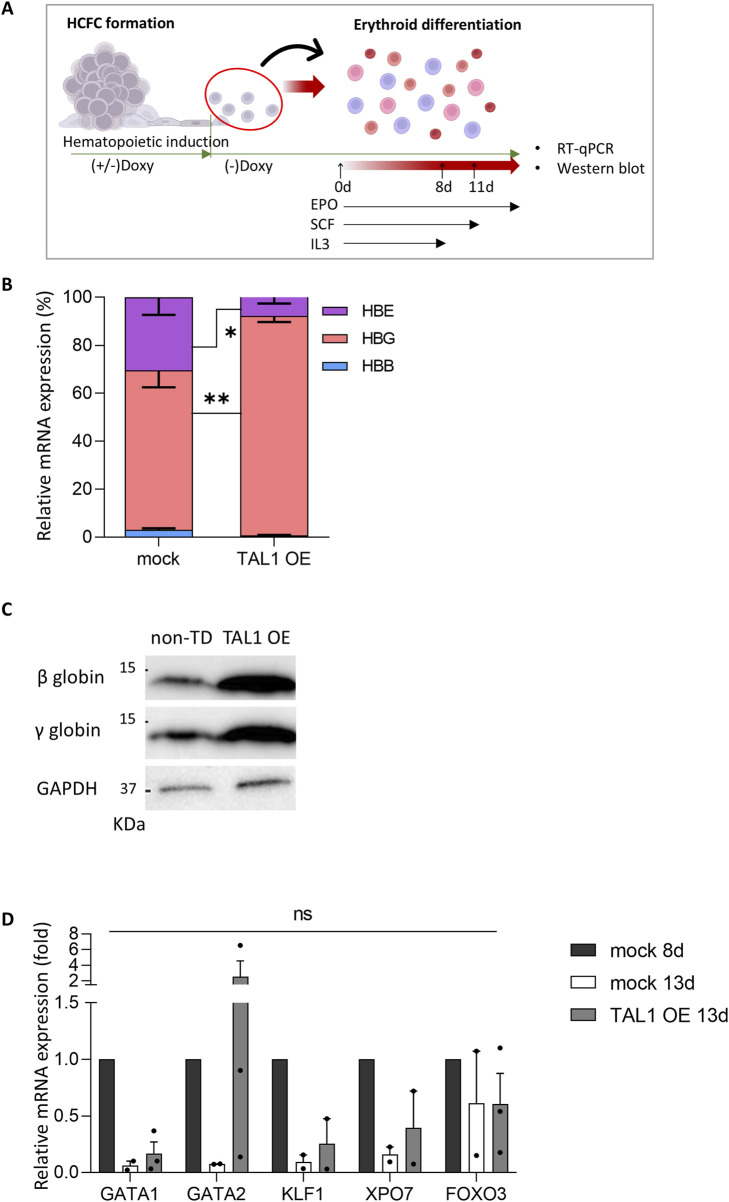
Expression of erythroid differentiation- and enucleation-related genes in Doxy (+) Tet-Tal1 iPSCs during terminal erythroid differentiation. **(A)** Schematic illustrating the experimental design. **(B)** The ratio of each globin mRNA in cells at day 8 of terminal differentiation (mock, n = 6; TAL1 OE, n = 4). *p < 0.05, **p < 0.01. **(C)** Expression of β- and γ-globin proteins in cells at day 13 of terminal differentiation. Western blots used for quantification are shown on the right side. A full membrane image of the Western blot is displayed in Supplementary Figure S7A. **(D)** The mRNA expression of differentiation- and enucleation-related genes (mock 8d, n = 2; mock 13d, n = 3; TAL1 OE, n = 3). Data are presented as relative quantification (2^−ΔΔCT^). Statistical analyses were performed using Wilcoxon’s signed-rank test for **(B)** and Student’s t-test for **(D)**. Data are shown as mean ± SEM.

Next, we examined the type of globin gene on day 8 of terminal differentiation in Doxy (+) Tet-TAL1 iPSCs (Figure 6A), although most cells were arrested at the orthochromatic stage. mRNA expression analysis revealed that the γ-globin gene (*HBG*) was the most prevalent in both mock and TAL1-OE groups, with *HBG* expression increasing to 91% in Doxy (+) Tet-TAL1 iPSCs. In contrast, β-globin (*HBB*) expression remained negligible, and ε-globin (*HBE*) expression was below 10% (Figure 6B). Furthermore, Western blot analysis on day 13 of erythroid differentiation demonstrated elevated levels of definitive globin proteins (β- and γ-globin) in Doxy (+) Tet-TAL1 iPSCs compared to non-transduced iPSCs (Figure 6C). These findings suggest that TAL1 overexpression enhances definitive globin protein production during hematopoietic induction but interferes with terminal erythroid differentiation.

To investigate the molecular mechanisms by which TAL1 OE inhibits terminal differentiation, particularly the enucleation step, we conducted a comparative analysis of the gene expression patterns of several genes (i.e., GATA1, GATA2, KLF1, XPO7, and FOXO3) associated with erythroid differentiation and enucleation in mock and TAL1-OE cells on day 13 of erythroid differentiation (Figure 6D). We used mock 8d as a positive control of immature erythroblasts to track gene expression patterns during terminal differentiation and compared the differentiation of mock 13d and TAL1 OE 13d.

Importantly, in the TAL1-overexpressing group, GATA2 expression remained high even after differentiation was complete. In contrast, in the mock group, GATA1 and GATA2 mRNA expression decreased as erythroid differentiation progressed. Meanwhile, the expression levels of KLF1, XPO7, and FOXO3 showed no significant differences between the mock and Doxy (+) TAL1-OE groups. The results of Wright–Giemsa staining and RT-qPCR show that the nuclei are condensed but not enucleated. These findings suggest that TAL1 OE during the hematopoietic induction disrupts terminal differentiation by inhibiting the transition from GATA2 to GATA1.

## 4 Discussion

RBCs are vital for transfusion and diagnosis. Challenges such as COVID-19 blood shortages, rising demand, safety concerns, and the need for specific phenotypes highlight the need for alternatives, including *ex vivo* and *in vitro* RBC production (Roberts et al., 2019; Chou et al., 2018). Recently, *in vitro* hematopoiesis using iPSCs has gained popularity because of the limited availability of hematopoietic stem and progenitor cells (HSPCs) and ethical concerns surrounding ESCs, which hinder large-scale RBC production. Unlike other cell sources, iPSCs are derived from somatic cells, have an unlimited supply, and differentiate into hematopoietic lineages, making them viable alternatives to HSPCs (Lu et al., 2008). Transitioning through the HSPC stage may be the ideal approach (Ju et al., 2024). Although this process is not yet fully understood, numerous efforts have been made to enhance the hematopoietic differentiation of iPSCs using various differentiation protocols in various culture environments. Differences in these differentiation methods result in variations in enucleation, expansion, and the type of hemoglobin expressed. This indicates that a distinct mechanism may occur in differentiating cells as they progress through the various stages of cell differentiation. Therefore, the transcription factors expressed during each differentiation process are distinct, potentially yielding disparate outcomes compared to those observed in previous studies.

To develop a promising strategy for improving RBC production from iPSCs, we evaluated the effect of conditional TAL1 OE on *in vitro* RBC production from iPSCs via HCFC formation. TAL1 OE enhanced HCFC formation, hematopoietic differentiation, and γ globin levels, evidenced by increased GPA+/CD71+ cells and hematopoiesis-related gene expression. We found that TAL1 OE during hematopoietic induction facilitated erythroid differentiation into more advanced erythroblasts, although other hematopoietic differentiations may also occur more efficiently. As TAL1 OE is a common cause of T-cell acute lymphoblastic leukemia (T-ALL) (Ferrando et al., 2002), it is not surprising that Doxy (+) Tet-TAL1 iPSCs exhibited higher differentiation into other hematopoietic cells. In contrast, we found that TAL1 OE during the hematopoietic induction stage enhanced GATA2 expression and the production of definitive globin proteins. The expression level of the γ-globin gene (*HBG*) was significantly increased at the expense of the ε-globin gene (*HBE*) expression on day 8 of terminal differentiation in TAL1 OE iPSCs. Furthermore, compared to non-transduced iPSCs, Doxy (+) Tet-TAL1 iPSCs exhibited higher levels of both definitive globin proteins (β- and γ-globin) on day 13 of erythroid differentiation. Our findings are consistent with previous studies showing that GATA2 and TAL1 promote γ-globin transcription (De Souza Carrocini et al., 2015) and that GATA2 OE enhances HBG expression during differentiation (Shearstone et al., 2016). Also, erythroid differentiation was delayed when γ-globin increased by downregulating the key regulator of fetal hemoglobin (Chumchuen et al., 2019). This may have resulted from the disruption of terminal differentiation by the inhibited transition from GATA2 to GATA1.

TAL1 OE in ESC-derived hemogenic endothelium has been shown to enhance differentiation into HSPCs (Serina Secanechia et al., 2022). This finding aligns with the previously *established in vivo* role of TAL1 and explains both the high rate of HCFC formation and the large number of cells collected weekly from a single HCFC in the TAL1-overexpressing group. A related study investigated the effects of TAL1 OE on immortalized iPSC-derived erythroid progenitor cells (HiDEPs) using OP9 feeder cells during differentiation into erythroid progenitor cells (Kurita et al., 2013). In HiDEPs, a decline in the cell survival rate was observed during terminal erythroid differentiation. In contrast, the present study demonstrated consistent survival rates throughout the differentiation process. Moreover, total TAL1 expression in this experiment tended to decrease as erythroid differentiation progressed, unlike in HiDEPs, where endogenous TAL1 expression increased. The discrepancy in the effects of TAL1 OE between these two systems may be attributed to the regulatory differences in the cell lines and differentiation methods used.

Erythropoiesis is a highly coordinated process regulated by the sequential activation and repression of genes, driven by a network of transcription factors, and modulated by epigenetic mechanisms. At each step, a finely tuned regulatory network ensures the precise activation of genes necessary for guiding cells to their specific fate.

Surprisingly, we found that TAL1 OE during hematopoietic induction impaired terminal erythroid differentiation, significantly reducing enucleation efficiency. TAL1 OE primarily arrested cells at the orthochromatic erythroblast stage, reducing the enucleation rate to approximately 15% compared to approximately 60% in the mock group. Although the nuclei of orthochromatic erythroblasts are condensed, impaired enucleation may result from mechanisms other than nuclear condensation, which is essential for enucleation (Mei et al., 2021). To date, no study has directly investigated the correlation between TAL1 expression and enucleation. Among the genes tested, *GATA2*, which is required for the expansion and survival of hematopoietic progenitors and stem cells, was identified as a potential inhibitor of enucleation.

Enucleation is a highly complex process that relies on mechanisms involving cell migration, endosomal trafficking, apoptosis, and unique cellular interactions within the microenvironment. Numerous factors, including cytoskeletal proteins, transcription factors, and microRNAs, have been implicated in various aspects of the dynamic enucleation process, such as chromatin condensation, cell cycle exit, nuclear polarization, reorganization of cell membranes and organelles, nuclear extrusion, and separation of pyrenocytes from reticulocytes. Recent reviews, such as those by Menon and Ghaffari (2021) and Newton et al. (2024), provide comprehensive overviews of these mechanisms.

Disruption of this balance can result in various hematopoietic disorders. The impairment of terminal differentiation and enucleation caused by TAL1 OE may stem from the intrinsic nature of TAL1 as a transcription factor. TAL1 plays a key role in the formation of transcriptional complexes (SCLs) with various factors including E47/E2A, LMO2, GATA1–3, Ldb1/2, ETO2, Runx1, ERG, and FLI1. Given that the timing and levels of TAL1 expression are crucial for orchestrating differentiation into specialized blood cells, the aberrant expression of genes encoding components of the SCL complex can lead to malignant states of blood cells (Vagapova et al., 2018).

In this study, we found that GATA2 expression decreased as erythroid differentiation progressed but remained high even after differentiation was complete in the TAL1-overexpressing group. Thus, TAL1 OE may inhibit terminal differentiation and enucleation, potentially via the upregulation of GATA2.

It is also important to note that TAL1-short (TAL1-s), a recently identified alternatively spliced form of the conventional TAL1 (TAL1-long; TAL1-l), plays distinct roles in hematopoiesis and cell growth (Sharma et al., 2023). The truncated TAL1-s protein, which lacks the ETO2-binding domain and phosphorylation sites but retains the DNA-binding and helix-loop-helix domains, is essential for erythroid progenitor differentiation. In contrast, the full-length TAL1-l protein is required for megakaryocytic differentiation of progenitor cells. In this study, we did not measure TAL1-s upregulation in the TAL1 OE group during differentiation. However, TAL1-s could be a promising candidate for enhancing RBC production from iPSCs *in vitro*.

In summary, TAL1 OE during hematopoietic cell induction promotes the differentiation of cells into HCFCs. However, it was also observed that the expression of additional transcription factors influenced the cells throughout the terminal differentiation phase, ultimately inhibiting enucleation. The reduced enucleation efficiency associated with TAL1 OE highlights a significant challenge that must be addressed to optimize the production of fully functional, transfusable RBCs. Therefore, it is essential to understand precisely how alterations in TAL1 protein expression during differentiation affect transcription factors at different times and how these changes lead to differences in differentiation and enucleation. Further research is required to balance the benefits of enhanced differentiation with the requirement for efficient enucleation, which is a critical step in the generation of mature and viable RBCs.

## Data Availability

The raw data supporting the conclusions of this article will be made available by the authors, without undue reservation.
