# Supplementary material for: TAL1 overexpression in induced pluripotent stem cells promotes the formation of hematopoietic cell-forming complexes but inhibits enucleation in vitro
Source: Front Cell Dev Biol. 2025 Apr 24;13:1474631. doi: 10.3389/fcell.2025.1474631 (PMC12058772; doi:10.3389/fcell.2025.1474631)
Supplement: Supplementary file 1 [file DataSheet1.pdf]

Supplementary Material

1 Supplementary method

1.1 iPSCs verification

iPSCs were cultured until the cell density reached approximately 50% confluence. The cells were then fixed in 4% paraformaldehyde (Biosesang, Seongnam, Korea) and permeabilized with 0.2% Tween 20 (AMRESCO, Pennsylvania, USA). Primary antibodies from the Embryonic Stem Cell Marker Panel (Abcam, Cambridge, UK) were used for staining. After washing with PBS, the cells were incubated with secondary antibodies: Alexa 594-conjugated goat anti-rabbit IgG (Invitrogen) and Alexa 488-conjugated goat anti-mouse IgG (Invitrogen). Nuclei were stained with DAPI, and the samples were mounted with a mounting solution for preservation (Figure S2A).

1.2 Three-germ layer differentiation

iPSCs were differentiated into the three germ layers using the STEMdiff™ Trilineage Differentiation Kit (StemCell Technologies). For mesoderm differentiation, 5×10^4 cells were seeded in a 6-well plate. For endoderm and ectoderm differentiation, 2×10^5 cells were seeded in a 6-well plate. Mesoderm and endoderm differentiation were carried out for 5 days, while ectoderm differentiation was performed for 7 days, each in their respective differentiation media. The differentiation media were changed daily. After differentiation, cells were fixed with 4% paraformaldehyde and then permeabilized with PBST for 10 minutes. For ectoderm, anti-Tubulin βIII (Santa Cruz Biotechnology, CA, USA); for mesoderm, anti-Brachyury (Santa Cruz); and for endoderm, anti-GATA4 (Santa Cruz) antibodies were diluted in 3% BSA in PBST and incubated at 4°C overnight (Varga et al., 2017). The secondary antibody was Alexa 488-conjugated goat anti-mouse IgG (Invitrogen) and was incubated for 1 h (Figure S2C).

1.3 Cell morphology

Cells were spread onto slides using a cytospin (Cellspin; Hanil Science Industrial, Incheon, Korea) and then stained with Wright-Giemsa to assess the degree of cell differentiation. Images were captured using an optical microscope (Nikon Eclipse TE2000-U; Nikon).

1.4 EB attachment rate measurement

The attachment rate of EBs was expressed as a ratio of the number of colonies attached to a well plate 3 weeks after hematopoietic induction, relative to the number of EBs initially placed in the well plate after EB formation.

1.5 Quantitative real-time PCR (qRT-PCR)

Total RNA was extracted from cells using Trizol Reagent (Ambion, Austin, USA). cDNA was synthesized using Superscript III First-Strand Synthesis System (Invitrogen). qRT-PCR was performed using SYBR green (TOPreal qPCR 2X PreMIX; Enzynomics, Daejeon, Korea) on an

Applied System 7500 (Thermo Fisher Scientific, MA, USA). Samples were normalized to GAPDH or HBG, and mRNA expression was analyzed using the ΔΔCt method.

1.6 Conventional PCR (PCR)

Total RNA was extracted from cells using Trizol Reagent (Ambion). cDNA was synthesized using Superscript III First-Strand Synthesis System (Invitrogen). PCR was performed using DiaStar Taq pol (Solgent, Deajon, Korea). The DNA was visualized after separation by 1.5% agarose gel electrophoresis (Figure S2B).

1.7 Flow cytometry

EBs were collected using a 5 ml pipet, treated with Gentle Cell Dissociation Reagent (StemCell Technologies), and incubated in a 37°C CO_2 incubator for 10 min. After washing with 1% bovine serum albumin (BSA), the cells were stained with antibodies Glycophorin A (GPA; CD235a)-PE/cy7 (Biolegend, California, USA) and CD71-APC (Biolegend). Flow cytometry analysis was performed using FACS Aria III (BD Biosciences).

1.8 Immunofluorescence

HCFC cultured in confocal dishes were fixed with 4% paraformaldehyde at room temperature and then washed with PBST (0.2% triton X-100 (AMRESCO) in PBS). CD34-PE/Dazzle 594 (Biolegend), CD43-FITC (Biolegend) were diluted in 3% BSA in PBST and incubated at 4°C overnight. After washing with PBST, the nuclei were stained with Hoechst 33342 (Invitrogen, 25 μg/ml) and treated with mounting solution for sample preservation. Images were captured using a confocal microscope (TCS SP5, Leica).

1.9 Western Blot

Cells were lysed in RIPA buffer supplemented with protease inhibitor (Sigma-Aldrich, Missouri, USA) and 5x sample buffer (GenDEPOT, Texas, USA). Cell lysates were separated by SDS-PAGE and transferred onto a PVDF (polyvinylidene difluoride) membrane (Milliopore, MA, USA). The membranes were blocked for 30 min in 5% skim milk. Primary antibodies were incubated overnight at 4°C, and secondary antibodies (Horseradish peroxidase-conjugated polyclonal goat anti-mouse or rabbit IgG H&L, 1:5000; Abcam) were incubated at room temperature. Target proteins were visualized using ECL solution (Thermo). The monoclonal primary antibodies used in this study were anti-GAPDH antibody (1:5000; Invitrogen #MA515738), anti-Hemoglobin β antibody (1:500; Santa cruz #sc-21757), anti-Hemoglobin γ antibody (1:500; Santa cruz #sc-21756), anti-TAL1 (1:1000; Cell signaling technology, MA, USA #12831s).

2 Supplementary Figures and Tables

2.1 Supplementary Figures

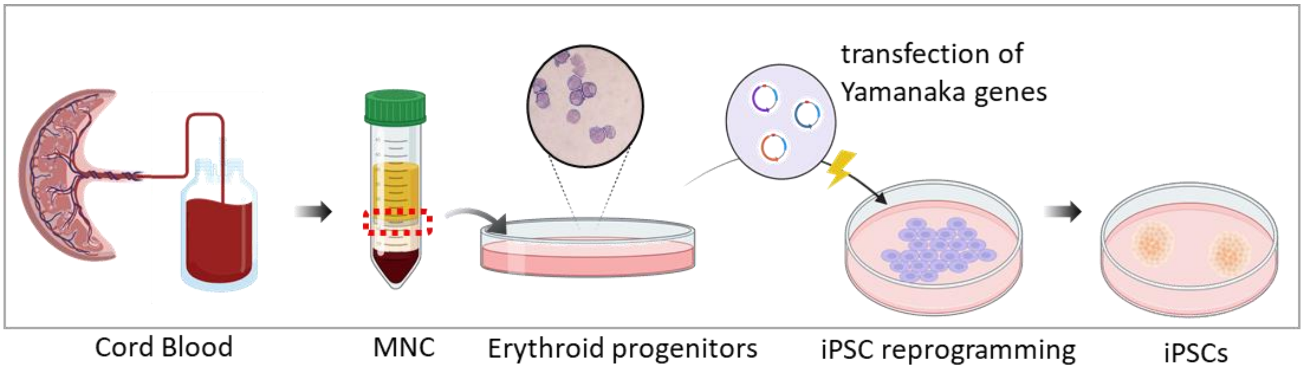

Supplementary Figure 1. Generation of CB-MNC-derived iPSC lines.
Donor-derived umbilical cord blood was centrifuged on Ficoll-Paque to obtain CB-MNCs. CB-MNCs were cultured for 6 days to differentiate into basophilic erythroblasts, with at least 80% differentiation. Erythroblasts were then electroporated with reprogramming vectors. The cells were cultured in reprogramming media for 21 days. Fully reprogrammed iPSC colonies were manually picked for further passaging.

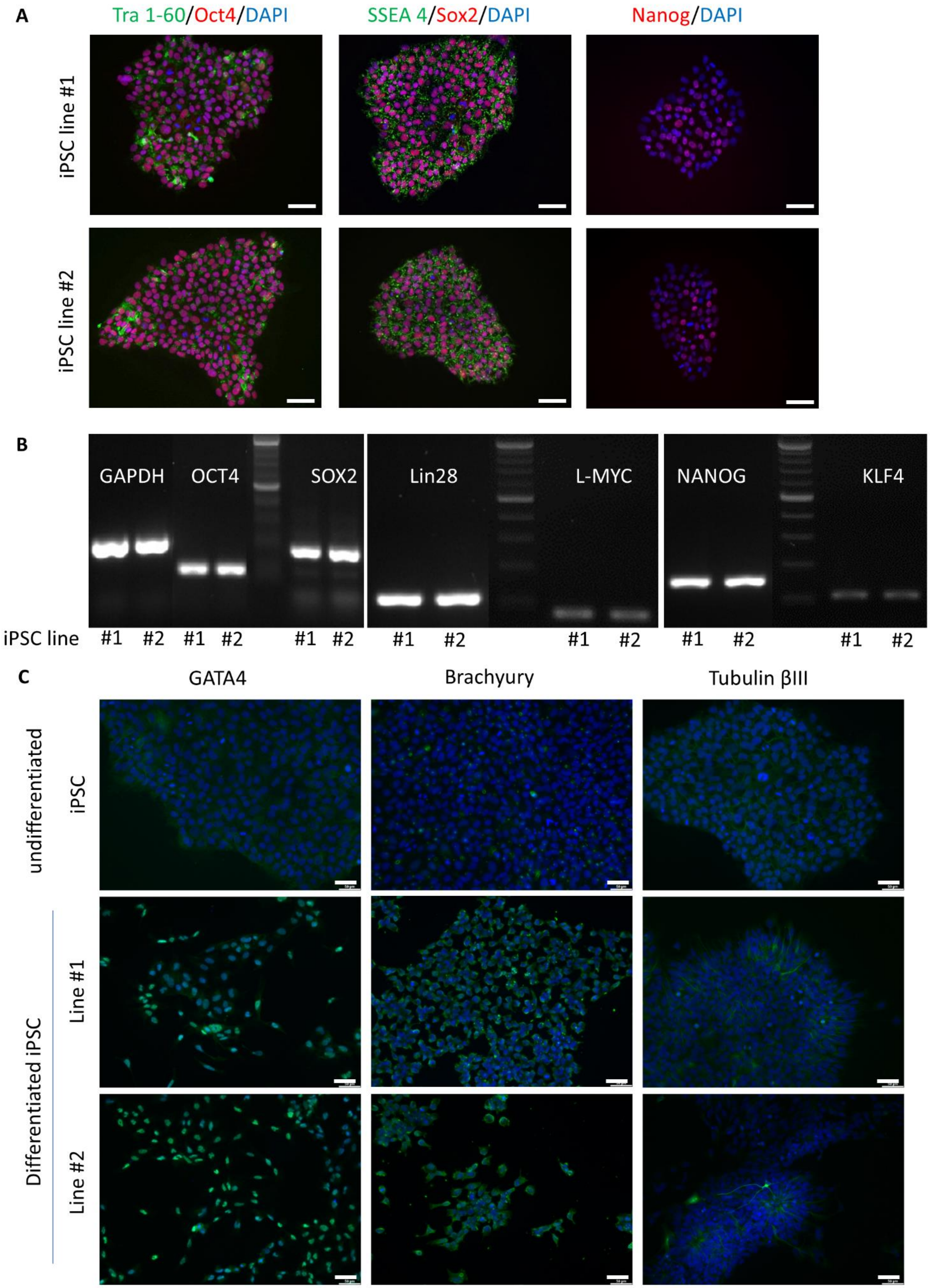
A
Tra 1-60/Oct4/DAPI
SSEA 4/Sox2/DAPI
Nanog/DAPI
iPSC line #1
iPSC line #2
B
GAPDH
OCT4
SOX2
Lin28
L-MYC
NANOG
KLF4
iPSC line
#1
#2
C
GATA4
Brachyury
Tubulin βIII
undifferentiated
iPSC
Differentiated iPSC
Line #1
Line #2

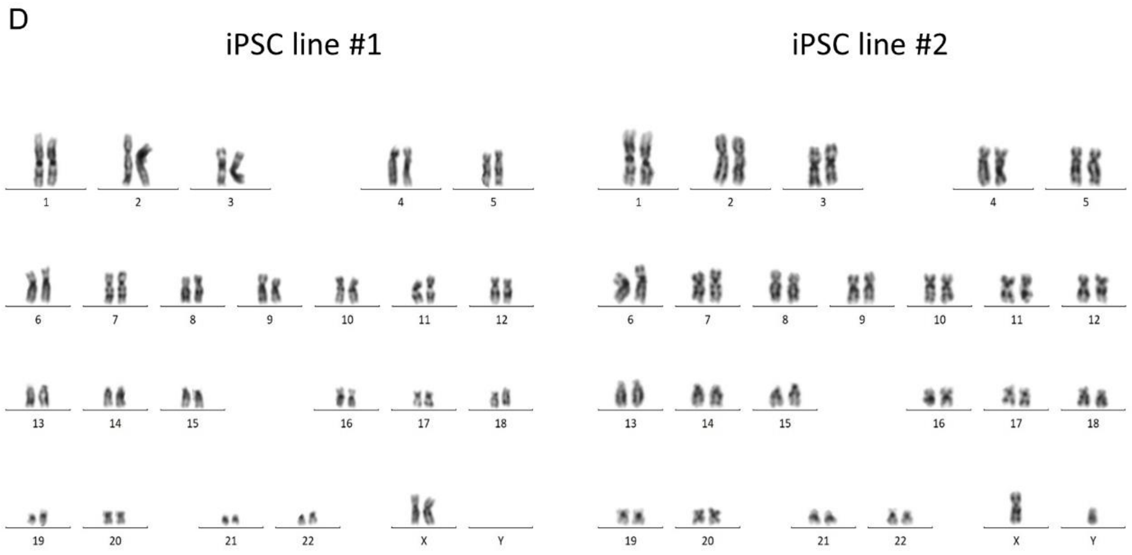

Supplementary Figure 2. Verification of the established iPSC lines.

(A) iPSCs were stained with an Embryonic Stem Cell Marker Panel, confirming the cells were fully reprogrammed. Scale bar = 75 µm; ×200. (B) Transfected genes were detected in iPSCs at passage 5 by conventional PCR. (C) iPSCs were differentiated using each differentiation media and stained with differentiation markers: ectoderm (Tubulin βIII), mesoderm (Brachyury), and endoderm (GATA4). Differentiated iPSCs expressed differentiation markers compared to undifferentiated iPSCs. (D) Karyotyping results of iPSCs showed a normal karyotype.

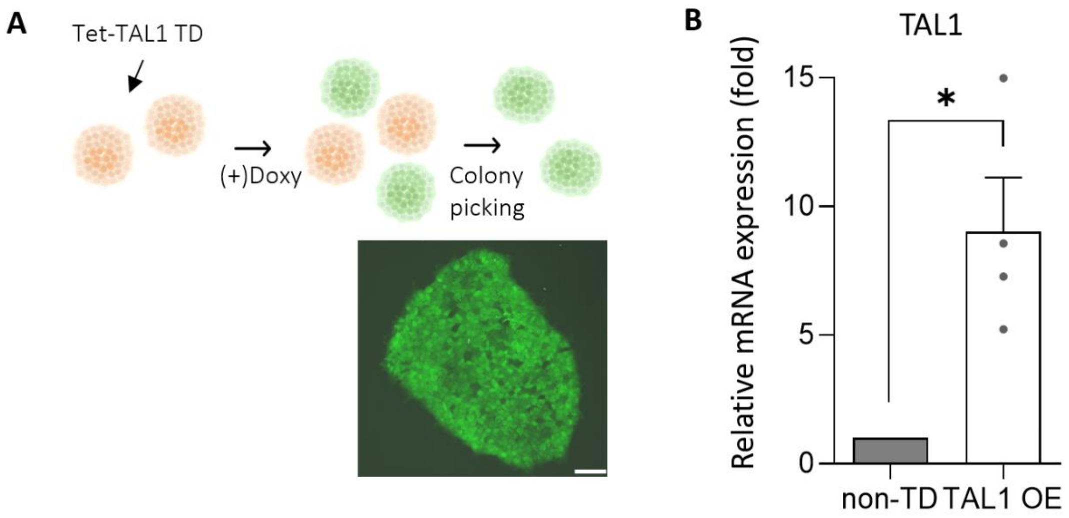

Supplementary figure 3. Isolation of TAL1 overexpressing iPSC colonies.

(A) Transduction of TAL1 lentivirus and manual picking of only GFP-positive colonies to obtain TAL1-overexpressing iPSC colonies. Scale bar = 100 µm. (B) Assessment of TAL1 overexpression in the isolated iPSC colonies by qRT-PCR (mock, n=8; TAL1 O/E, n=4). *$p < 0.05$.

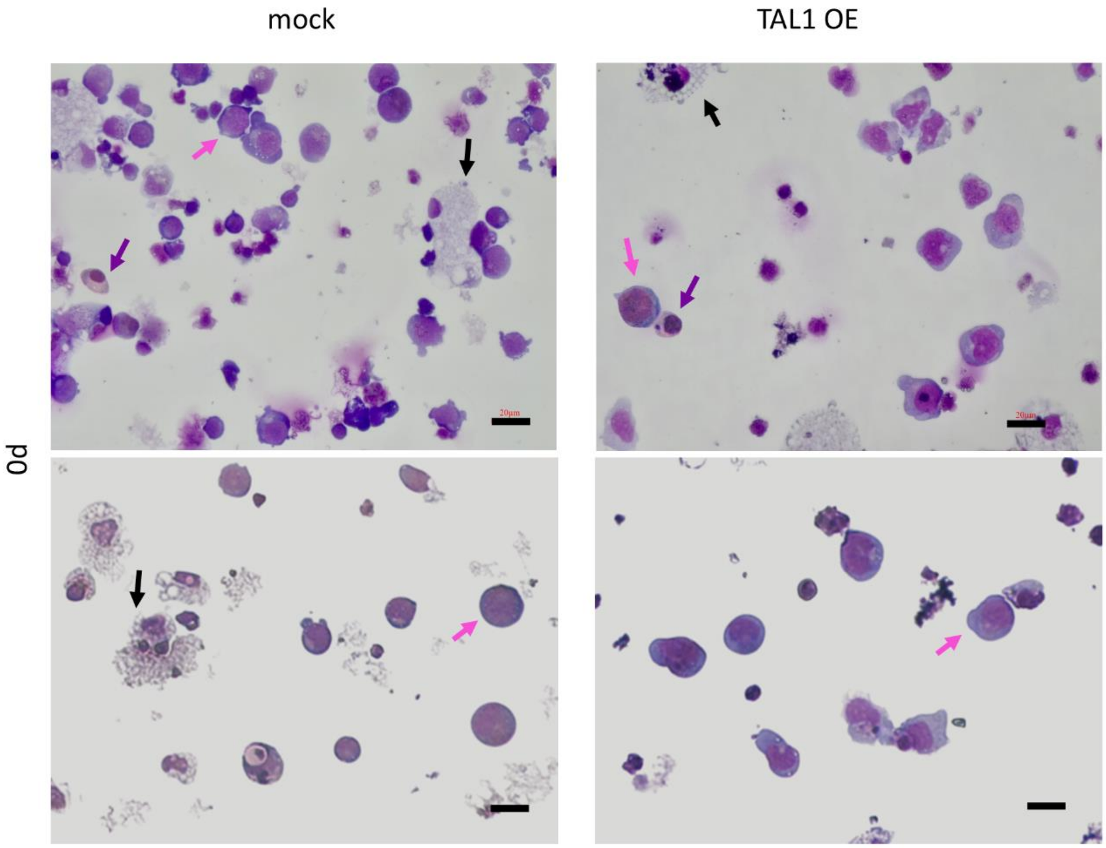

Supplementary figure 4. Photographs of Wright–Giemsa stained cells released from HCFCs.

Representative photographs show Wright–Giemsa-stained erythroid lineage cells released from week 5 HCFCs. Scale bar = 20 μm. Pink arrows indicate proerythroblast/basophilic erythroblasts, purple arrows indicate polychromatic erythroblasts, and black arrows indicate non-erythroid cells.

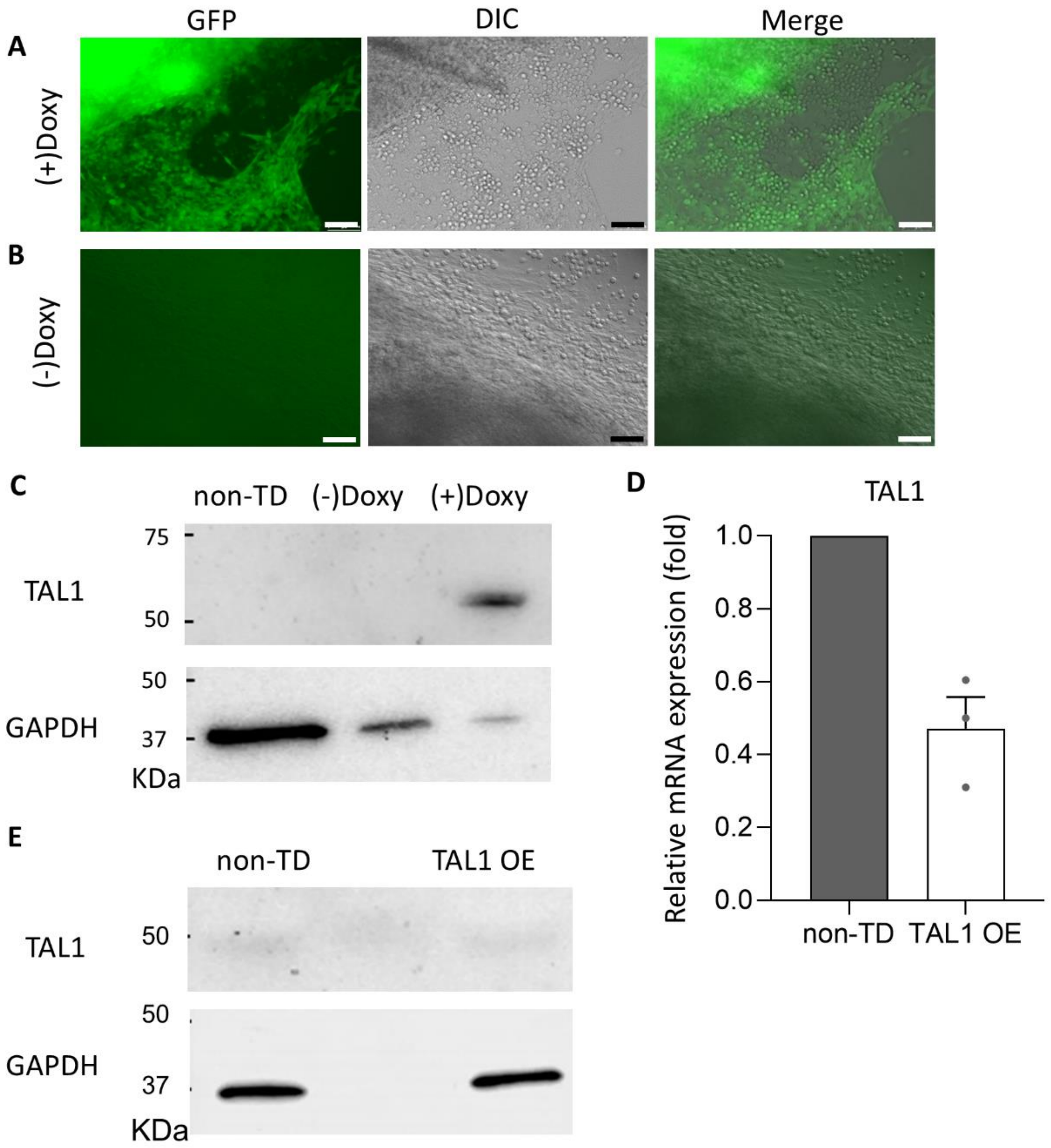

Supplementary figure 5. Estimation of doxycycline-dependent TAL1 OE in Tet TAL1 iPSCs under various culture conditions.

(A) GFP fluorescence visualization in iPSCs in the presence of doxycycline. (B) GFP fluorescence visualization in iPSCs 2 weeks after removal of doxycycline from the culture medium. (C) TAL1 is not expressed in non-doxycycline-treated and non-TD samples in Western blot analysis of iPSCs. (D) Relative mRNA expression of total TAL1 was measured after 8 days of erythroid differentiation (mock, n=4; TAL1 O/E, n=4). (E) After 13 days of erythroid differentiation, TAL1 expression is rarely detected, regardless of TAL1 OE, in Western blot analysis. Full membrane images of western blot were displayed in Figure S7.

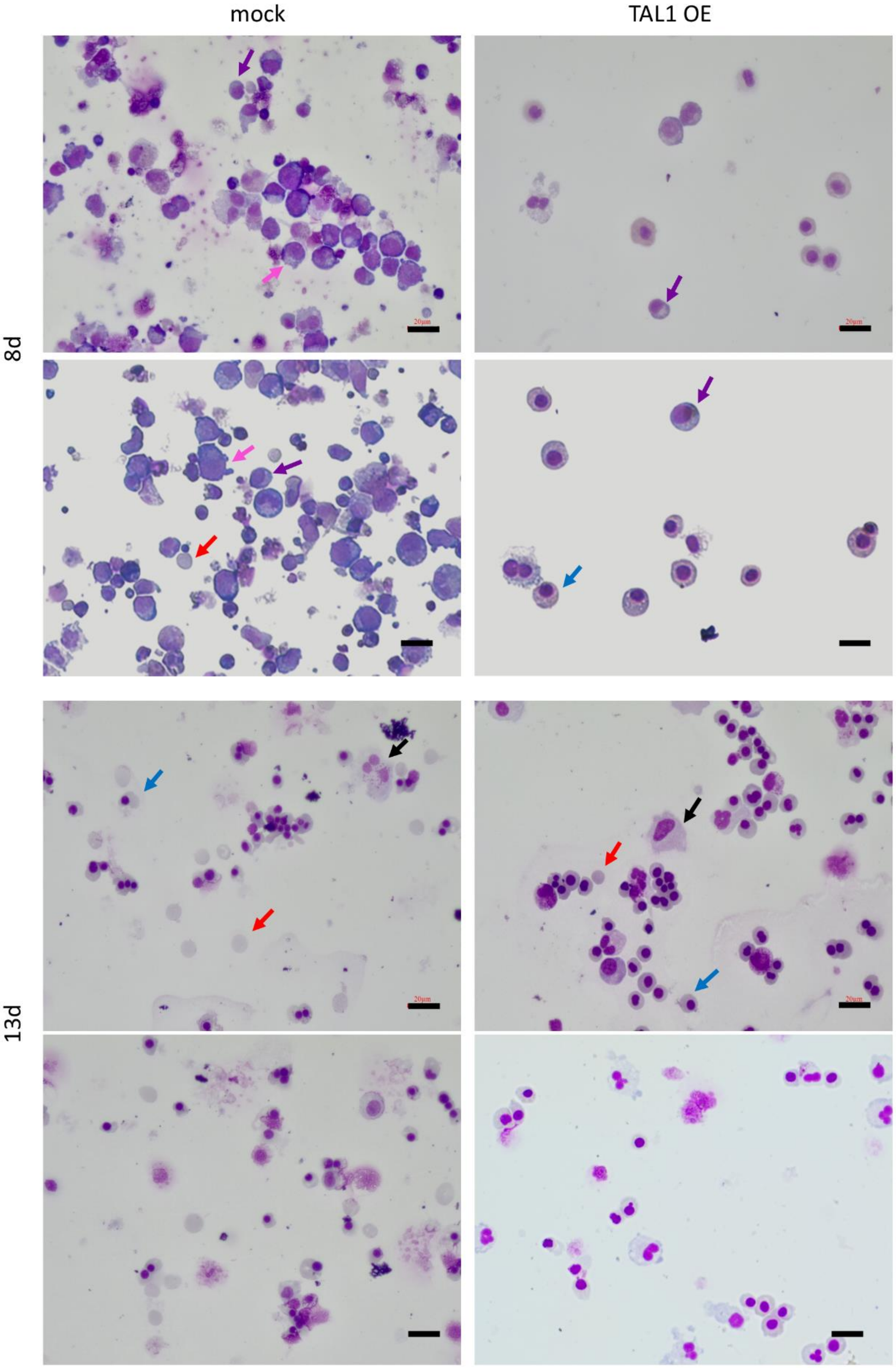

Supplementary figure 6. Representative photographs of the Wright–Giemsa stained cells.

Representative photographs show Wright–Giemsa-stained erythroid lineage cells at day 8 and day 13 after induction of terminal erythroid differentiation. Scale bar = 20 μm; ×200. Pink arrows indicate basophilic erythroblasts, purple arrows indicate polychromatic erythroblasts, red arrows indicate reticulocytes, blue arrows indicate orthochromatic erythroblasts, and black arrows indicate non-erythroid cells.

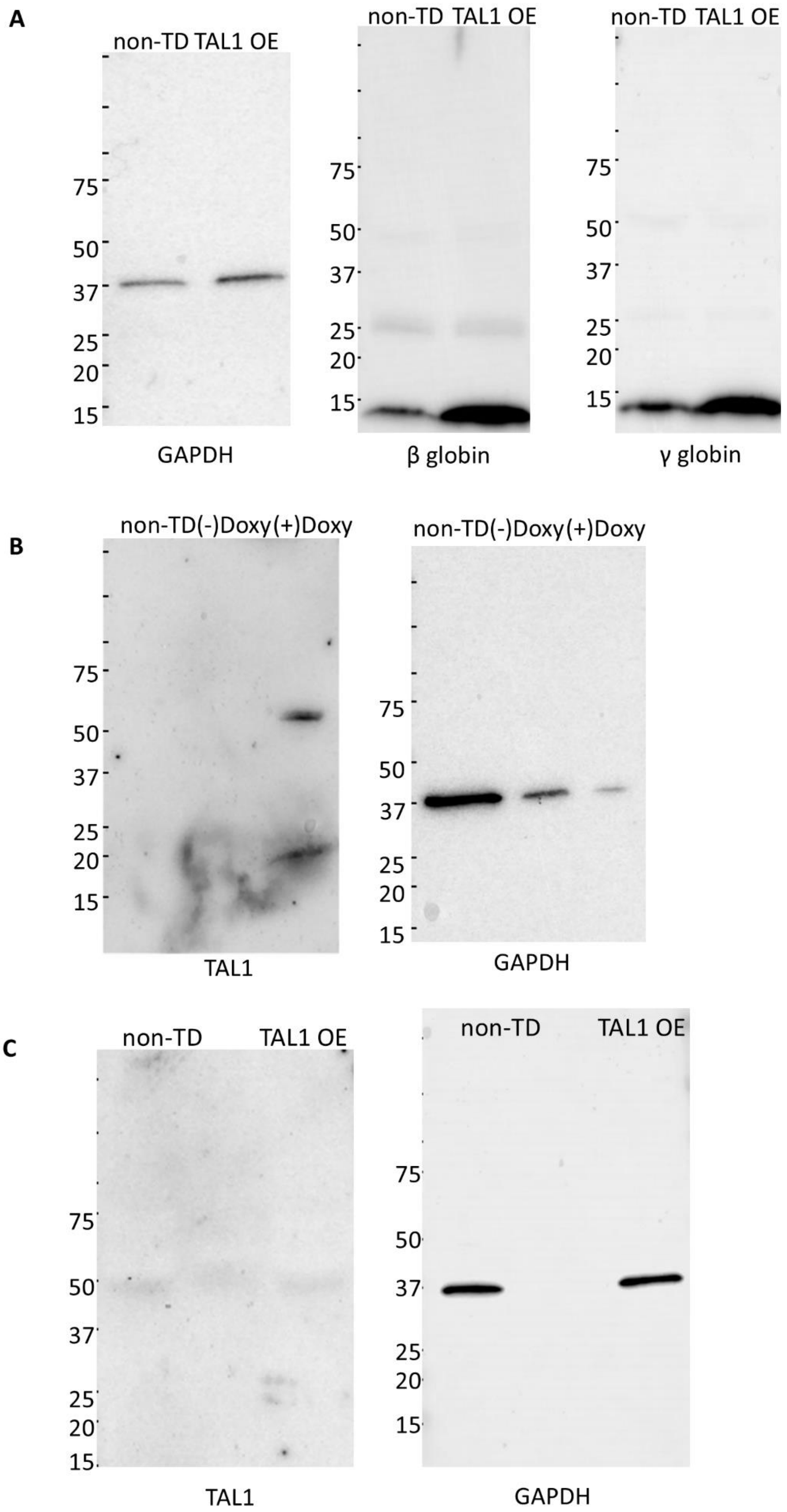
A
non-TD TAL1 OE
75
50
37
25
20
15
GAPDH
non-TD TAL1 OE
75
50
37
25
20
15
β globin
non-TD TAL1 OE
75
50
37
25
20
15
γ globin
B
non-TD(-)Doxy(+)Doxy
75
50
37
25
20
15
TAL1
non-TD(-)Doxy(+)Doxy
75
50
37
25
20
15
GAPDH
C
non-TD
TAL1 OE
75
50
37
25
20
15
TAL1
non-TD
TAL1 OE
75
50
37
25
20
15
GAPDH

Supplementary figure 7. Full membrane image of Western blot.

(A) Full membrane image of Figure 6C. (B) Full membrane image of Supplementary figure 5C. (C) Full membrane image of Supplementary figure 5E.

References

VARGA, E., HANSEN, M., WUST, T., VON LINDERN, M. & VAN DEN AKKER, E. 2017. Generation of human erythroblast-derived iPSC line using episomal reprogramming system. *Stem Cell Res*, 25**,** 30-33.
